# Quality of Life in Patients With Cushing's Disease

**DOI:** 10.3389/fendo.2019.00862

**Published:** 2019-12-11

**Authors:** Alicia Santos, Eugenia Resmini, Mª Antonia Martínez Momblán, Elena Valassi, Luciana Martel, Susan M. Webb

**Affiliations:** ^1^Endocrinology/Medicine Departments, Hospital Sant Pau, Centro de Investigación Biomédica en Red de Enfermedades Raras (CIBERER, Unidad 747), IIB-Sant Pau, ISCIII and Universitat Autònoma de Barcelona, Barcelona, Spain; ^2^Fundamental and Medico-Surgical Nursing Department, School of Medicine and Health Sciences, University of Barcelona, L'Hospitalet de Llobregat, Spain

**Keywords:** quality of life, health related quality of life, Cushing's syndrome, Cushing's disease, persistent morbidity

## Abstract

Cushing's disease (and by extension, Cushing's syndrome) is a rare disease due to a chronic cortisol excess, which usually has an important impact on quality of life (QoL). It can lead to numerous comorbidities that can interfere with daily life, as fatigability, myopathy, bone loss and fragility, increased cardiovascular risk, depression, and cognitive alterations. Of note, psychological alterations (including depression and anxiety) occur often, and are an important determinant of impaired quality QoL. QoL scores using different questionnaires are poorer in comparison to healthy controls, other pituitary adenomas and some chronic diseases. Even if some improvements can be observed after successful treatment, recovery does not seem to be complete, and comorbidities persist. This persistent QoL impairment has been found using both generic and disease-specific QoL questionnaires, and is also reported by the patients themselves, when asked directly. Multidisciplinary teams are essential to improve patients' well-being. Clinicians should take into account the whole scope of clinical problems and address the different comorbidites associated with the disease. Screening in the psychological sphere, with further intervention if necessary, can be helpful in the management of these patients. Interventions and programs have shown promising results, although there is a need for further development of new strategies for the benefit of these patients.

## Introduction

Endogenous Cushing's syndrome (CS) is a rare disease due to a chronic cortisol excess. It affects predominantly women, with a female/male ratio ranges from 3 to 5 (1). Even if the cause of hypercortisolism may vary (being of pituitary, adrenal or ectopic origin), pituitary origin (also called Cushing's disease -CD) is the most common cause of endogenous CS, affecting approximately 70% of the patients (2). This paper will deal mainly with CD, although some references to relevant studies in CS with a high proportion of patients with CD are also included.

Comorbidities caused by CD can have an important effect on QoL. Some of the symptoms determine physical changes that can easily be perceived by others and may affect patient's social interactions. These include facial plethora, weight gain, central obesity, supraclavicular fat accumulation, hirsutism, acne, purple striae, easy bruising, poor wound healing and ulceration; and also growth retardation in case of children (3). Furthermore, CD can also involve some morbidities that may not be easily perceived externally, but greatly affect patients' daily life. These include sleep disturbances, fatigue, myopathy, hypertension, alterations of glucose homeostasis (including diabetes mellitus and insulin resistance), dyslipidemia, prothrombotic state, vascular disease, atherosclerosis, menstrual and libido disturbances, hypogonadism and body composition alterations, including increased fat mass, reduced lean mass, and decreased bone mass leading to osteoporosis (3). Additionally, brain studies have also shown functional and structural alterations in the central nervous system (4). Psychological disturbances may also be present, including depression, anxiety, emotional irritability, apathy and cognitive decline, which may also affect family relationships (3, 5–10).

It is important to mention that in clinical practice a wide variety of features are referred by the patients. In the early stages of the disease, they may experience very few symptoms. In other patients it may take years to find the cause of their discomfort (11). In fact, according to data from the European Register on Cushing's syndrome (ERCUSYN), the median delay to diagnosis in patients with CD is 2 years (12). Delay to diagnosis often leads to more pronounced symptoms, which affect daily life, especially when they include psychological disturbances (as anxiety or depression) or troublesome symptoms, as fatigue. This can interfere in social and family relationships, as well as in their professional performance (11). This latter is particularly important, as some patients may be unable to work, with important effects on their income. Data from the ERCUSYN database report that only 47% of the patients were actively employed, despite a mean age of 44 years (12).

Even if symptoms usually improve after hormonal normalization, some impairment may remain. The recovery is slow and not all symptoms fully reverse. In fact, patients in remission may still have a persistent increased cardiovascular risk, body composition alterations and depression, anxiety or impaired cognition (6, 13–15). Furthermore, pituitary deficits requiring hormonal substitution can show up after treatment. Therefore, patients usually complain of incomplete recovery, both physical and psychological, affecting their QoL (16, 17).

It is essential to perform periodic, life-long evaluations to properly manage persistent comorbidities in order to improve patient's outcome (17). This also includes QoL and other psychological aspects, which impact on the well-being and even survival of the patients. Of note, depression in patients in remission has been found to increase the hazard of death, and elevate the risk of cardiovascular disease (18). Furthermore, it is also important to consider the perspective of the patient in order to be able to understand and approach all problems globally that CD may involve. An interesting study reported that 71% of the patients felt that CS had affected their lives greatly, while 20% chose “a lot.” No patients chose “not at all” or “very little” (8). Therefore, it would be important to consider patient-reported outcomes during follow-up and to provide appropriate multidisciplinary treatment if necessary.

## Quality of Life and Health Related Quality of Life: The Concepts

Being a complex concept, different definitions of QoL have been developed. According to the World Health Organization (WHO) QoL can be defined as “an individual's perception of their position in life in the context of the culture and value systems in which they live and in relation to their goals, expectations, standards and concerns” (19). In other words, QoL could be considered a patient-reported measure that reflects their individual definition of well-being (including patients' expectations and physical, emotional and social aspects) (20).

QoL is a widely-used concept that has received growing interest by clinicians and researchers in the last decades. However, when dealing with the effects on QoL that diseases can have, some authors prefer to use a more specific concept: health-related QoL. It could be defined as “those aspects of self-perceived well-being that are related to or affected by the presence of disease or treatment” (21).

## How Can Health-Related Quality of Life Be Measured?

Many instruments exist in order to measure health-related QoL. When choosing a tool the essential criterion is to select one that is validated and has good psychometric properties. Questionnaires are the most commonly used tools to measure health-related QoL, as they are easy to use and administer in both clinical practice and research. Many questionnaires exist to measure health-related QoL, and they can be classified as generic and disease-specific.

Generic questionnaires can be used in patients with any disease or even in healthy individuals, which is helpful to make comparisons between different populations. Therefore, it can be interesting to use this kind of questionnaires if we want to compare patients with healthy controls, or if we want to compare patients with different kinds of pituitary adenomas. Some common generic questionnaires are Short-Form 36 (SF-36), Short-form 12 (SF-12) or Nottingham Health Profile. However, when dealing with a specific disease, this kind of questionnaires may not approach some particular aspects of the disease (20).

In contrast, disease-specific questionnaires have been developed to evaluate specific QoL aspects of a certain disorder. They are more sensitive to identify specific impairments related to a disease, but as a limitation they can only be used in that specific disease. Therefore, they are more useful for longitudinal analysis in a population with a specific disease (for instance, in the follow-up of a particular patient) or to compare different patients with the same disease (17, 20).

In the case of CD, there are two disease-specific questionnaires available: CushingQoL and Tuebingen CD-25. CushingQoL was the first disease-specific questionnaire for CS and is a short questionnaire consisting in 12 questions with a 5-options Likert scale answers, with demonstrated good psychometric properties (22–24). It was initially considered unidimensional, although a later analysis has identified two subscales: psychosocial issues and physical problems (25). Of note, a recent study has demonstrated that interpretation of three of the items categorized under psychological issues can vary between different nationalities, making it important to take this into account for interpretation (26). The other questionnaire was developed for patients with CD: the Tuebingen Cushing's disease quality of life inventory (Tuebingen CD-25). This questionnaire consists in 25 questions and includes 6 different domains: Depression, Sexual Activity, Environment, Eating Behavior, Bodily Restrictions and Cognition (27, 28). In this questionnaire lower scores indicate a better QoL (in contrast with CushingQoL). Less studies are available with this questionnaire, although some psychometric properties had been demonstrated, as high reliability and validity, sensitivity to change and construct and criterion validity (27, 29, 30). A strong correlation has been found when comparing scores of both CushingQoL and Tuebingen CD-25 (R = −0.733) (30).

There are also other types of questionnaires that may not be directly considered QoL questionnaires, although they are able to measure a specific domain which considerably impacts on QoL. These include comorbidities as depression, anxiety, fatigue, sexual function, sleep, pain, or self-steem (20). Table 1 summarizes different questionnaires that can be used to assess health-related QoL in patients with CD.

**Table 1:** Different questionnaires that can be used to assess quality of life in patients with Cushing's disease/syndrome.

		**Questionnaire list**
Generic quality of life questionnaires		Notingham Health Profile (NHP) Short Form-36 (SF-36) Short Form-12 (SF-12) (SF-36 in a short form) Psychological General Well Being Scale (PGWBS) EuroQoL-5D (EQ-5D) World Health Organization Quality of Life BREF (WHOQoL-BREF)
Disease-specific quality of life questionnaires		CushingQoL Tuebingen Cushing's disease quality of life inventory (Tuebingen CD-25)
Specific domains:	Depression	Hospital Anxiety and Depression Scale (HADS) Beck Depression Inventory-II (BDI-II)
	Anxiety	Hospital Anxiety and Depression Scale (HADS) Beck Anxiety Inventory (BAI) State Trait Anxiety Inventory (STAI)
	Fatigue	Checklist individual strength Questionnaire (CIS) Modified Fatigue Impact Scale (MFIS)
	Self-steem	Rosenberg's questionnaire
	Sexual function	Female Sexual Function Index (FSFI) International Index of Erectile Function (IIEF-5)
	Pain	Mc Gill Pain Questionnaire
	Fatigue	Checklist Individual Strength Questionnaire
	Sleep	Pittsburgh Sleep Quality Index (PSQI) Insomnia Severity Index

## What Are the Effects of Cushing's Syndrome on Health-Related Quality of Life?

**Figure 1:**
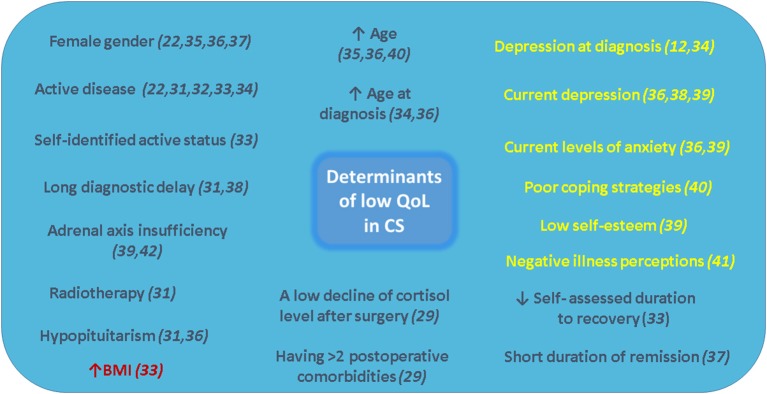
Determinants of low quality of life in Cushing's syndrome. Modifiable factors using specific interventions have been highlighted (yellow for psychological determinants and red for BMI).

It is now clear that CS and CD have a deleterious effect on the QoL of the affected patients, although the extent may vary from one patient to another. Different determinants have been described that impact on quality of life (12, 22, 29, 31–42). A summary can be found in Figure 1.

There are important differences in QoL between patients with active disease and patients in remission of CD. Therefore, two separate sections to analyzing QoL during the different phases of the disease are presented.

## Effects of Active Disease on Quality of Life

Quality of life is clearly impaired in patients with active disease. In CD QoL is worse than in controls and patients with any other kind of pituitary adenomas (35, 43). QoL is similarly impaired in pituitary-dependent CD and other causes of CS, as shown in a cohort of patients from different European countries included in the ERCUSYN database using CushingQoL and EuroQoL (12, 34).

SF-36 results are low in physical and mental summary scores compared to healthy controls matched for age and sex (35, 44). When compared to other untreated pituitary tumors (which not have been treated) CD were the patients with most impairment in all SF-36 measures (43). This impairment is especially relevant if patients do not achieve remission after treatment (specifically, scores <25th percentile for age and sex in most of the SF-36 domains) (45). Negative correlations have been found between 24 h urinary free cortisol and the mental component score of SF-36 (but not with the physical component score) in patients with CD (32).

Medical treatment can be helpful to improve QoL in active disease. In a sample of 162 patients treated with pasireotide, a mean improvement in CushingQoL of 11.1 points was found after 12 months (46). Further analysis of the sample revealed that QoL improvements were associated to lowering of urinary free cortisol levels, reduction of both BMI and weight, and improvement in depression (47). Twenty-four-week treatment with mifepristone in CS has also been shown to improve the mental and physical scores of SF-36 and depression, measured by BDI-II (48). In a small sample of 17 patients using different drugs sequentially (pasireotide, ketoconazole or cabergoline) during 80 days, no statistical changes in QoL were found when compared to basal values (no improvement or worsening). However, improvement was found after 1 year in 3 patients that continued medical treatment, suggesting that QoL improvements after medical therapy may be found in the long run, but not so much short-term (49).

Depression is typically found in patients with CD and is suggested to be an early manifestation of CS (3, 50). At the disease onset (or even before diagnosis) it is present in up to 25% of the patients, and the percentage may increase over time if the disease is not treated (10). Data from the ERCUSYN have reported it in 38% of the patients at diagnosis (12). When using specific diagnostic criteria, major depressive disorder has been reported in 50–80% of the active patients according to different studies, with higher presence in the older studies (10). Furthermore, the level of depression correlates with QoL scores measured with SF-36 in female patients with CS, who are clearly worse than controls (44).

Anxiety is also more prevalent in CS than in healthy population, as evidenced with questionnaires like STAI (51). It seems to be more prevalent in pituitary-dependent CD that in other causes of CS (52). One study using specific diagnostic criteria found that generalized anxiety disorder was present in 79% of the patients, while panic disorder was found in a variable range from 3 to 37% of the patients, possibly associated with a more chronic stage of the disease (10, 53, 54). Other psychiatric disorders reported include mania or hypomania (3–30% of the patients) and psychotic disorders (8% of the patients, but more common in adrenal carcinomas, where it was found in up to 40% of the patients) (10, 55–57). Obsessive-compulsive disorder has also been observed in children (3).

Cognitive alterations are also commonly found in patients suffering from CD. Memory is the cognitive function most frequently studied, with impairment in both verbal and visual memory (51, 58–62). Although less consistently, impairment has also been found in other cognitive functions like executive functions, visoconstructive skills, language, motor functions and information processing speed (63).

This poor cognitive performance appears to be related to specific brain specific alterations. For instance, smaller hippocampal volumes have been found in active patients than in controls, which have been related to learning and memory dysfunction, and also to cortisol blood levels (64). Adult patients with active disease also have smaller cerebellar cortex and right amygdala volumes than healthy controls (51, 65). Reduced amygdala volumes in comparison to controls have also been found in children with CS (66). Left amygdala volumes are negatively correlated with depression and anxiety scores in active CS (but not in patients in remission) (65). Interestingly, right cerebellar cortex volumes correlated with QoL scores, while left cerebellar cortex volumes correlated with visual memory (51). Other brain dysfunctions present in active CS include changes in the concentration of different brain metabolites like the choline/creatine ratio on the frontal and thalamic areas (67), diffuse alterations in white matter integrity suggesting demyelization (68) and altered activation in different brain structures important for emotion perception (69).

Finally, other specific symptoms may have an important impact in patient's lives. Many suffer from insomnia, specifically middle insomnia (59%), late insomnia (57%), or early insomnia (29%) (70). Furthermore, although sexual function has not often been investigated in CD, it is frequently impaired. Reduced libido is reported in 50–69% of patients with active CS (10, 12, 55, 70). A study in a sample of 29 sexually-active women with active CS (89.7% with CD) showed that female sexual dysfunction was present in 88.9% of the patients. Scores of the Female Sexual Function Index (FSFI) were lower than in healthy controls. More specifically, lower scores for arousal, lubrication, orgasm, satisfaction and higher levels and frequency of pain during intercourse were found, while no differences were found for sexual desire. FSFI scores were positively correlated with LH levels (which were lower in the patient sample compared with controls) (44).

QoL is also impaired in children and adolescents. Female to male prevalence decreases with younger age, and in fact in prepuberal children the disease seems to be more prevalent in males (71). Children with CD can experiment personality changes, which typically include compulsive behavior, moodiness, irritability and overachievement in school (72). Some authors have reported altered amygdala and hypocampal function during a faces encoding task (which assesses emotional encoding and memory), in adolescents with CS, which is not related to memory and affective systems, in contrast with findings in adult patients (73).

## Effects of Remission in Quality of Life

Patients in remission of CS have better QoL than active patients measured with both disease-specific and generic questionnaires (23, 35). The origin of the disease does not seem influence QoL, which was similarly impaired in patients with CS, irrespective of the cause (37). After successful treatment, improvement often takes months or over a year, while the immediate postoperative phase can be troublesome, including more pain, fatigability and a poor perception of QoL (22). This in part may be due to the glucocorticoid withdrawal syndrome, which occurs after successful surgery. If the patient is not aware of this, the first weeks after surgery can prove very cumbersome and worrying. A study on patients after adrenalectomy (including 28% of patients with CD) found that mean time for symptom resolution was at least 7 months, and some symptoms as acne took up to a mean of 17 months to resolve (74). In fact, not all patients perceived themselves as fully recovered. For instance, in a cohort of 102 patients treated for CD 92% of the patients reached criteria for biochemical cure, while only 80.4% perceived themselves as in remission (33).

Significant improvement from baseline values can be detected with CushingQoL 4 months after successful transsphenoidal surgery (23). Using Tubingem 25-CD in a sample of 17 patients with CD, improvement was found after surgery in most of the domains, including sexual activity, environment, eating behavior, bodily restrictions and cognition. However, no improvement was found for depression. Predictors for improvement in QoL after surgery were preoperative QoL impairment (meaning that patients with poorer QoL preoperatively were more likely to improve post surgery) and age (meaning that younger patients were more likely to improve post surgery) (29). Another study reported impairment in the 6 dimensions of Tuebingem 25-CD questionnaire in patients treated for CD (85% in remission after surgery) (75). In addition, women were more affected than men for the dimensions of eating behavior and cognition of the Tuebingem 25-CD questionnaire (40).

Improvements in QoL have also been reported using generic questionnaires in patients with CD after transsphenoidal surgery. More specifically, using SF-36, improvements were found for bodily pain, vitality, social functioning, role limitations due to emotional health, general health, mental health and mental summary scores; while no improvement was found for physical functioning, role limitations for physical health and physical summary (35). SF-36 scores in patients surgically treated for CD have showed more impairment in women, specifically for physical functioning, physical role functioning and emotional role functioning (40).

Negative illness perceptions in patients in remission of CS are another feature which negatively affect QoL. Correlations between the Illness Perception Questionnaire Revised (IPQ-R), and QoL questionnaires (CushingQoL and the visual analog scale of EuroQoL) have reflected that a worse QoL is related to affected illness perceptions (41).

However, improvement in QoL after remission is not complete in most of the cases, and some degree of impairment tends to persist after cure (35, 36, 76). Table 2 reflects a list of the most common symptoms still present after surgical remission in patients with CS. Patients in remission still have poorer QoL scores than healthy controls. Generic questionnaires have shown impairment in comparison to healthy population and normative values using different questionnaires, like the Nottingham Health Profile (36), WHOQoL-BREF (39) or both mental and physical summary scores of SF-36 (35, 36, 75). In addition, patients in long-term follow-up of CD show impaired physical functioning, measured with SF-36, when compared to patients with non-functioning adenomas (77). CushingQoL scores are associated with depression, anxiety, cortisol insufficiency and self-steem (39). In a big cohort of patients included in the ERCUSYN database, 1 year after treatment patients with CD pituitary origin had worse QoL than those of adrenal origin. However, in a regression analysis etiology was not related to QoL after adjustment for baseline age, gender, remission status, duration of active CS, glucocorticoid dependency and follow-up time. Furthermore, when different symptoms at diagnosis were included in a regression analysis (hypertension, diabetes mellitus, muscle weakness, and depression) only depression was associated with a worse CushingQoL score at last follow-up visit (34).

**Table 2:** Most common symptoms still present in patients in remission of CS after surgical remission [adapted from Lindsay et al. (35); sample of 343 patients with a mean time since treatment of 11.8 years].

**Symptom**	**Percentage of patients reporting the symptoms (%)**	**Symptom**	**Percentage of patients reporting the symptoms (%)**
Fatigue	41.3	Bulging abdomen	29.3
Forgetfulness	35.7	Anxiety	28.4
Trouble sleeping	33.3	Facial hair	27.6
Depression	31.2	Feelings of sadness	27.6
Weight gain	30.4	Mood swings	27.4
Decreased muscle strength or weakness	30.4	Decreased ability to exercise	27.0

The presence of persistent comorbidities can also have an important role on patient's quality of life. In fact, having more than two postoperative comorbidities has been reported as a predictor of low quality of life (29). These may include hypertension, dyslipidemia, central obesity or persistent diabetes mellitus, among others, leading to an increased cardiovascular risk (13). In patients treated for Cushing's syndrome, increased BMI has been reported as predictor of poorer quality of life (measured with CushingQoL) (33). Control of certain comorbidities like diabetes may require specific additional chronic medication and lifestyle changes, which may influence patient's perception of well-being.

Self-assessment of the remission status provides some interesting information, and may be discordant with the biochemical status. In a cohort of 102 patients treated for CD, CushingQoL scores were higher in patients that perceived themselves as in remission when compared to patients that perceived themselves as having persistent disease. This group that perceived themselves as biochemically cured also had lower scores in anxiety and depression, measured with HADS. When patients with real biochemical remission were analyzed, those who perceived themselves as cured had better QoL than patients who did not feel “cured,” although they also had a longer duration of follow-up. The authors suggested that this may reflect a discordance between biochemical and psychological recovery times, psychological recovery being slower. On the other hand, patients who were hypercortisolemic had poorer QoL scores and higher depression scores compared to patients who were biochemically cured (33).

Patients perceive that their body changes dramatically after onset of the disease, and that it never returns to its original size. However, no clear relationship was found between QoL and characteristics of a drawing test, were patients drew themselves before, during and after CS treatment (78). Patients in remission of CS also showed a lower body satisfaction in comparison with normative values measured with the Body Satisfaction Score. Body satisfaction is also associated with the social relations domain of the WhoQoL questionnaire. In the same study, 61.9% of the patients had a low or very low self-steem (39).

Hypopituitarism contributes to worse QoL in patients treated for CD. In a longitudinal study of 58 patients who had undergone transsphenoidal surgery (and radiotherapy in 19% of the patients), hypopituitarism was present in 52% of the patients, and was an independent predictor of worse QoL (36). In line with these findings, a study of 99 patients with CD and 24 patients with CS of adrenal origin found that both groups had impairment in most of the QoL dimensions compared to controls (with no differences between both patient groups). However, when pituitary patients were analyzed, patients without any pituitary deficiencies had better QoL scores (specifically in CushingQoL and the energy item of the NHP questionnaire) and also in the items of fatigue and motivation form the Checklist Individual Strength Questionnaire (a questionnaire designed to measure fatigue) when compared to patients with deficiencies. Even if impairment was still present in comparison to controls, in the group without pituitary deficiencies it was only found in half of the dimensions evaluated (37). Other studies have also found higher fatigue in CS patients in remission than in healthy controls, measured with MFI-20 and MFS (36, 79).

Patients with CD that underwent bilateral adrenalectomy, compared to patients who underwent other treatments, had poorer QoL, measured with SF-36 (mainly in bodily pain, general health, vitality and social functioning) and CushingQoL. They also presented worse depressive scores measured with the BDI. The major predictor of poor QoL was adrenal insufficiency (42). This is in line with a prior study, where even if patients reported improvement in their QoL after bilateral adrenalectomy, scores from SF-36 were all lower that the general population (80).

Different studies have described higher depression and anxiety levels in comparison with literature values, healthy controls and non-functioning adenomas, measured with different questionnaires, including HADS, BDI, BDI-II and or STAI (36, 37, 51, 75, 81, 82). Increased age and male gender have been related to less anxiety levels in a cohort of patients treated by transsphenoidal surgery for CD (33). Current depression scores, measured with BDI-II and CES-D, have been associated to delay to diagnosis in patients in remission of CS (38). Patients had higher scores than controls for depression, anxiety, stress perception and QoL. Interestingly, levels of blood brain-derived neurotrophic factor (usually expressed in brain areas that control mood and stress response), were related to higher depression, anxiety and stress, and to decreased affective balance (38).

Another study found that patients in remission of CD presented higher depression, anxiety, apathy and irritability than healthy controls. Depression (measured with HADS) was positively influenced by remission duration; while irritability (measured with the Irritability Scale) was related to radiotherapy, and on the other hand apathy (measured with the Apathy Scale) was associated by level of education. Those patients also presented maladaptative personality traits, with poorer performance in several scales of the Dimensional Assessment of Personality Pathology short-form in comparison with controls. Of note, when anxiety and depression were controlled as covariates only differences in affective liability and anxiousness remained statistically significant (81). In another study from the same group it was demonstrated that patients with pituitary adenomas had less effective coping strategies than healthy controls, although patients with CD sought more social support than patients with non-functioning adenomas (83). In a focus group study including 6 patients with CD some maladaptive coping strategies (as overdoing or problems with acceptance) were identified as possible contributors of low QoL (84). In fact, a further study found that maladaptative coping strategies (mainly depressive coping, trivialization, and wishful thinking) were correlated to different measures, indicating a relationship with poorer QoL (measures with both Tuebingem CD-25 and SF-36) and higher depression, anxiety and embitterment (40). Furthermore, QoL scores were also related to embitterment (measured with the Bern embitterment questionnaire-BEI). Some degree of embitterment was found in more than a half of the patients compared to reference values. Specifically 36.8% of the patients had a medium degree of embitterment, 18.7% had an above-average degree of embitterment and 3.5% had an extreme degree of embitterment (40)

Cognitive function, including memory and executive functions, remains impaired after cure of CD. This has been found 1 year after biochemical cure (85, 86), 3 years after surgery for CS (9) and also in patients with long-term remission, with lower scores when compared to healthy controls and non-functioning adenomas (6, 82, 87, 88). One study in CS in long-term remission reported that cognitive alterations were independent of concomitant fatigue and affective disorders, while etiology was not related to cognitive symptoms (82).

In line with findings for cognitive function, brain alterations may also persist in patients in remission. They include altered functionality (as changes in the concentrations of different brain metabolites or loss of white matter integrity due to decreases of axial diffusivity) (89–93) and structure (4, 7, 88), with lower brain volumes than controls in adults (affecting total volumes and gray matter) (51). Subjective loss of brain volume can also be found in 86% of the patients with CD after cure (94). In contrast, in children reduced brain volumes seem to reverse after cure (66). Some authors report a slight increase of the hippocampus and right caudate volume after cure of CD (95, 96). Hippocampal volume increase was also related to word learning improvement (95). Other studies in patients in remission of CS have found no differences in hippocampal volumes, when compared to healthy controls (7, 97), although smaller hippocampal volumes were found in patients with severe memory impairments (97). Of note, brains of patients in remission show a higher degree of white matter lesions (visible in the MRI) when compared to controls and patients with active disease. The severity of white matter lesions has been correlated to duration and degree of hypertension, reflecting the increased prevalence of cardiovascular risk factors in CS. Furthermore, lower gray and total brain matter volumes have been found when compared to normal controls, and brain volume was correlated to 10-year cardiovascular risk (6).

Even after surgery children may also still experience low QoL, although improvements may be found in comparison to levels during active disease (98). In a sample of children and adolescents, incomplete recovery of the hypothalamic-pituitary-adrenal axis 1 year post-treatment was related to poorer QoL in physical and psychosocial domains, measured with a generic health-related QoL questionnaire (the child health questionnaire-parent report). Furthermore, changes in physical function scores were inversely associated with BMI (98). Most of the persistent comorbidities found in adults, as altered body composition, can also be found in children (99). However, brain function and volume seems to be affected by cortisol excess in a different way when compared to adults. In fact, a recovery of brain volume has been described 1 year after cure, with a decrease in cognitive function and a decline in school performance, with no associated psychopathology (66). Brain plasticity in these younger patients may contribute to these findings which differ from the experience in adults. Furthermore, in children with CS, final height is very often compromised, lower than midparent height (100).

## Hints to Improve Patient's Quality of Life

Clinicians play an important role in helping patients to improve their QoL. First of all, it is important to treat all comorbidities. As reported before, some modifiable factors can have a great impact in QoL (as depression, anxiety, poor coping strategies, low self-steem, negative illness perceptions or high BMI). Patients should be asked for their symptoms and needs and referred to specialists if necessary, including psychologists and/or psychiatrists. It would also be important to inform the patients on the persistent adverse events that CS may have on their QoL, so inappropriate expectations can be avoided (77). Giving printed information may be helpful, so the patients can have time to analyze and review it if necessary. Additionally, an empathic relationship is essential, as in fact the doctor-patient relationship can have an important impact on patient outcome, and even survival (101).

Patients with CS of any cause may benefit from educational interventions. A 9-month educational nursing program in patient groups with CS (most of them in remission) demonstrated improvements in sleep patterns, pain, healthy lifestyle and physical activity, which positively influenced QoL. This program consisted on 4 educational sessions (2 of them including also family members) and one follow-up visit. The topics included basic information of CS, management of comorbidities, self-care, practice of balanced diets and recommendations for healthy lifestyle (102). In this line, the Endocrine Society Guidelines recommend educating patients with CS on the clinical features of remission of CS, but also their families (103). Given the low prevalence of the disease (approximately 39.1 patients per million inhabitants) patients may feel very lonely and not understood (104). Therefore, support groups can also be helpful to help patients deal with CS and its consequences. Many options exist, which do not need to be face-to-face (facebook, whatsapp or websites can be used). Clinicians can facilititate contacts to the patients if local groups are available. Finally, maintaining healthy habits and lifestile is also important, positively influencing patient outcome and well-being (10).

A study on German and US patients with CS in remission (only CD for Germany) explored patient's needs through a questionnaire. Patients reported that good medical care and skilled doctors where what helped them most to cope with the disease (48.8% US, 44.4% Germany), followed by support from family, friends and religion (36.9% US, 31.7% Germany). Regarding educational and support programs, most of the patients preferred internet-based programs (89.3% US, 75.4% Germany). However, cultural differences and specific needs may differ in different populations of patients with CS (105). In other chronic diseases programs that improve self-manegement (where the patient has a more active role) have lead to better health status and increased QoL (105). Consequently, it would be helpful to develop specific programs for patients with CD too.

In conclusion, health-care providers can use different strategies to improve patient's well-being. Screening and management of persistent comorbidities, including psychological aspects, is essential. Education on the disease and what to expect can be very helpful, as well as an empathic attitude, to make the patient aware that the symptoms, signs and limitations he or she is experiencing may be related to the prior diagnosis of CS, and can be approached to improve their day-to-day outcome. Group programs have shown promising results, but still few are available, so this is a pending issue for the future.

## Author Contributions

AS wrote the manuscript. ER and MM helped with the literature review and editing. All authors revised the manuscript.

### Conflict of Interest

The authors declare that the research was conducted in the absence of any commercial or financial relationships that could be construed as a potential conflict of interest.
